# Supplementary material for: A high-resolution mRNA expression time course of embryonic development in zebrafish
Source: eLife. 2017 Nov 16;6:e30860. doi: 10.7554/eLife.30860 (PMC5690287; doi:10.7554/eLife.30860)
Supplement: Supplementary file 6. [file elife-30860-supp6.zip › biolayout-clusters-files/Cluster188-genes.html]

Cluster188


# Cluster188: Genes

| Ensembl ID | Gene Name | Chr | Start | End | Biotype |
| --- | --- | --- | --- | --- | --- |
| ENSDARG00000015709 | hsd17b12a | 25 | 29753032 | 29787552 | protein\_coding |
| ENSDARG00000104278 | slc2a8 | 5 | 65291120 | 65307489 | protein\_coding |
| ENSDARG00000054423 | slc7a6 | 7 | 28354532 | 28376766 | protein\_coding |
| ENSDARG00000058780 | slc9a2 | 9 | 6984610 | 7013259 | protein\_coding |
| ENSDARG00000061963 | vrtn | 20 | 27850043 | 27855924 | protein\_coding |
| ENSDARG00000074617 | wfs1b | 14 | 17024242 | 17045506 | protein\_coding |
| ENSDARG00000088967 | wnk4b | 3 | 22312765 | 22416759 | protein\_coding |
